# How to build a human

**DOI:** 10.7554/eLife.19826

**Published:** 2016-08-24

**Authors:** Carla B Mellough, Majlinda Lako

**Affiliations:** Institute of Genetic Medicine and North-East England Stem Cell Institute, Newcastle University, Newcastle upon Tyne, United Kingdom; Institute of Genetic Medicine and North-East England Stem Cell Institute, Newcastle University, Newcastle upon Tyne, United Kingdommajlinda.lako@ncl.ac.uk

**Keywords:** transcriptome, organogenesis, embryo, RNA-Seq, Human

## Abstract

The genes that control the development of specific tissues and organs in human embryos have been identified.

**Related research article** Gerrard DT, Berry AA, Jennings RE, Hanley KP, Bobola N, Hanley NA. 2016. An integrative transcriptomic atlas of organogenesis in human embryos. *eLife* **5**:e15657. doi: 10.7554/eLife.15657

**Image** Like barcodes, patterns of gene expression can be used to identify specific cells types, tissues and organs

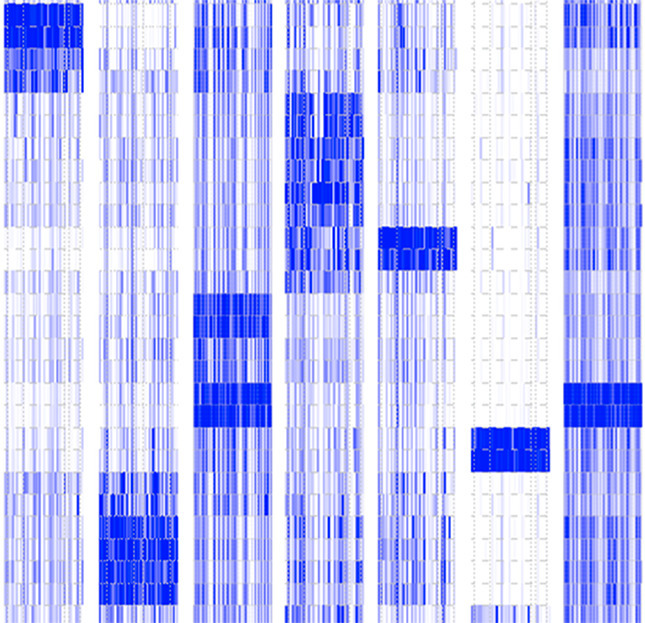

The manual for human development is written in the genetic code of our DNA. Our genes encode the instructions to make specific types of molecules that are needed to either build the human body or to control its day-to-day operations. The first step in producing these molecules involves the gene being copied, or transcribed, into an RNA molecule. For some genes, this RNA molecule is the end product; for other genes, the RNA molecule must then be translated to produce a protein.

Mistakes, or mutations, in a gene often change the RNA or protein produced, and even the smallest of changes can have far-reaching effects. For example, if these mutations affect molecules that are needed when a human embryo is developing in the womb, they can lead to severe abnormalities that are collectively called congenital disorders. A cleft palate is a relatively common example of a congenital disorder, and affects one in every 700 babies born each year (Forrester and Merz, 2004; Parker et al., 2010). Similarly, mutations that result in genes being active at the wrong time, or in the wrong cells in a developing embryo, can also lead to congenital disorders. In addition, exposure to toxic or infectious agents can also be a cause. This is exemplified by the drug thalidomide, which was administered to pregnant women between 1956 and 1962, and has caused severe birth defects in around 10,000 children worldwide (McBride, 1961).

Understanding why organs sometimes form incorrectly is challenging, largely because tissue from developing human embryos is rarely available for study. Instead, research has tended to extrapolate information from multiple sources, including individuals born with specific disorders, animal models and cell-based laboratory models of development and disease (Azamian and Lalani, 2016; Moretti et al., 2013; Kelly, 2016).

Some studies have investigated which genes are expressed in developing human embryos by looking at the RNA molecules produced at a given time. These “transcriptomics” studies, however, have tended to look at the embryo as a whole (Fang et al., 2010), or to focus on specific organs and tissues in more developed embryos (van den Berg et al., 2015; Slieker et al., 2015). Consequently, little was known about the formation and early development of organs – a process called organogenesis – in humans.

Over the past year or so, data on this crucial process has started to emerge. First, in mid-2015, a study described the transcriptional profiles of human embryos from different developmental stages (Roost et al., 2015). Now, in eLife, Neil Hanley and co-workers – from the University of Manchester and the Central Manchester University Hospitals NHS Foundation Trust – report transcriptomics data obtained during human organogenesis (Gerrard et al., 2016). The human embryonic material used in the study was collected according to the Codes of Practice of the UK Human Tissue Authority (HTA, 2016).

Hanley and co-workers – who include Dave Gerrard and Andrew Berry as joint first authors – looked at the RNA molecules found in organs and tissues from fifteen separate sites in human embryos. These sites included the thyroid, liver, stomach, brain, heart and adrenal gland. Gerrard et al. were then able to integrate this data into a sort of atlas that mapped the activity level of developmental genes across the embryo. This in turn allowed them to identify eleven groups of genes that were expressed differently in the different organs and tissues examined. Gerrard et al. refer to these groups of genes as metagenes and the activity levels of genes found in each metagene form a kind of transcriptional barcode for each of the different tissue types of the body.

Many cell-based models of development use stem cells from human embryos, or other cells that have been reprogrammed to be more like stem cells (so-called induced pluripotent stem cells; Takahashi et al., 2007). However, these approaches have their limitations (Hrvatin et al., 2014; Patterson et al., 2012), and it is challenging to ensure that stem cells differentiate into completely mature tissues in vitro. Gerrard et al. confirmed that the transcriptional barcode associated with the embryonic liver (metagene 2) was also seen in liver tissue derived from stem cells grown in the laboratory. This suggests that the transcriptional barcodes could be used to check that lab-grown stem cells have differentiated and matured as intended. Moreover, knowing which genes actively direct the cells in embryos to differentiate in specific ways could help other researchers to coax stem cells to become a wider range of tissues in vitro.

The work of Gerrard et al. adds substantially to what is known about the transcriptional profiles of each of the organs and tissues in a developing human embryo, and about the disorders associated with these tissues. Furthermore, their findings complement what we know about the later stages of embryonic development (Roost et al., 2015), and will allow a greater understanding of the distinct events that occur during human development and disease.

The findings by Hanley, Gerrard, Berry and colleagues have great implications for improving stem cell research. They will also undoubtedly aid research into diseases that develop at later stages of development, which has proved particularly challenging so far. Might we be on the cusp of completing the last pages of the ‘how to build a human’ manual? If so, soon we may finally be able to generate mature tissues more routinely in vitro and unravel the unknown mechanisms of development and disease.
